# A Quasi-Steady-State Approximation to the Basic Target-Cell-Limited Viral Dynamics Model with a Non-Cytopathic Effect

**DOI:** 10.3389/fmicb.2018.00054

**Published:** 2018-01-31

**Authors:** Richard A. Cangelosi, Elissa J. Schwartz, David J. Wollkind

**Affiliations:** ^1^Department of Mathematics, Gonzaga University, Spokane, WA, United States; ^2^Department of Mathematics and Statistics, Washington State University, Pullman, WA, United States; ^3^School of Biological Sciences, Washington State University, Pullman, WA, United States

**Keywords:** quasi-steady-state approximation, viral dynamics, equine infectious anemia virus, HIV, dynamical systems, matched asymptotic expansion

## Abstract

Analysis of previously published target-cell limited viral dynamic models for pathogens such as HIV, hepatitis, and influenza generally rely on standard techniques from dynamical systems theory or numerical simulation. We use a quasi-steady-state approximation to derive an analytic solution for the model with a non-cytopathic effect, that is, when the death rates of uninfected and infected cells are equal. The analytic solution provides time evolution values of all three compartments of uninfected cells, infected cells, and virus. Results are compared with numerical simulation using clinical data for equine infectious anemia virus, a retrovirus closely related to HIV, and the utility of the analytic solution is discussed.

## 1. Introduction

**Figure 1:**
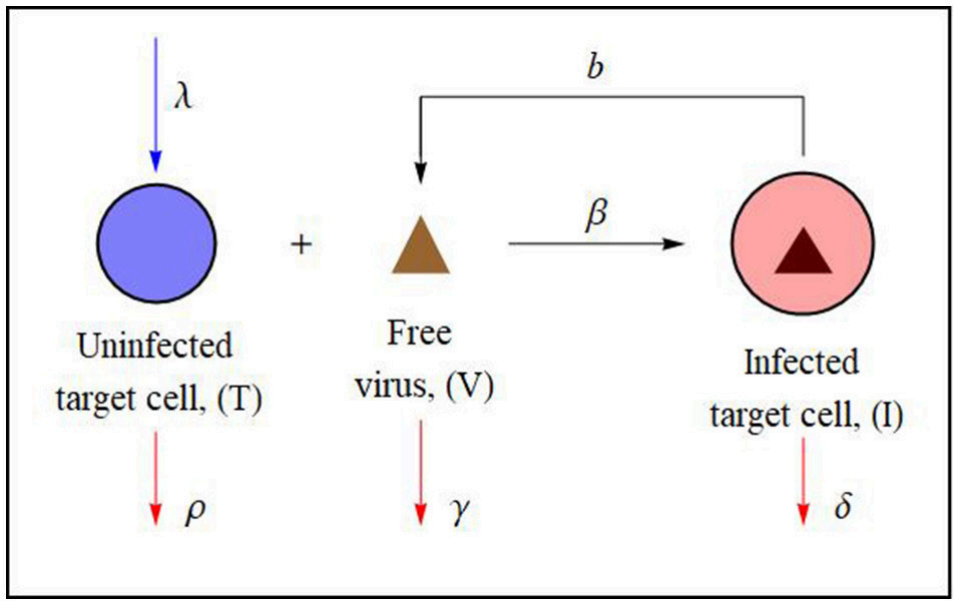
Schematic diagram of the basic target-cell-limited viral dynamics model illustrating cell-virus interactions. Uninfected target-cells (T) can be infected by the virus (V) to create productively infected cells (I) (see e.g., Perelson and Ribeiro, 2013). In the case of a non-cytopathic virus ρ ≈ δ. The associated mathematical model (Equation 1) is described and analyzed in section 2.

Mathematical models have proven valuable in understanding the dynamics of viral infections *in vivo* within host cells and were originally devised to examine HIV infection (reviewed by Perelson and Ribeiro, 2013). For interactions of that sort, a basic three-component dynamical systems model consisting of an uninfected target-cell population, an infected cell population, and the free virus population was proposed (see Figure 1). This model implied that the propagation of the virus was limited by the availability of susceptible target-cells and hence is now characterized as target-cell-limited (Phillips, 1996). Assuming a rapid enough time-scale for the free virus dynamics so that a quasi-steady-state approximation could be employed, Tuckwell and Wan (2004) formally reduced this basic target-cell-limited viral model system to a two-component one consisting of the uninfected and infected target-cells. They then showed that there were no periodic solutions for the two-component model and that the trajectories of both systems remained quite close. DeLeenheer and Smith (2003) and Prüss et al. (2008) studied the global stability of the biologically relevant equilibrium points for this basic target-cell-limited viral model system and found that its behavior depended upon the size of a particular non-dimensional parameter *R*_0_, the basic reproductive number, to be defined in the next section. If *R*_0_ < 1, they demonstrated that the virus-free equilibrium point was globally asymptotically stable, while if *R*_0_ > 1, this property shifted to the disease-persistence equilibrium point.

The results cited above use either standard techniques of dynamical systems theory or numerical simulations. Defining α as the ratio of the death rates of the infected to the uninfected cells, Burg et al. (2009) classified such viral interactions to be either cytopathic or non-cytopathic depending upon whether α > 1 or α = 1, respectively. During cytopathic viral interactions the infected cells are killed by the virus during the course of infection. Some viruses are intrinsically non-cytopathic because they replicate in a relatively benign manner while others actively maintain such a state by shutting down all destructive processes, activating non-destructive mechanisms, or inducing alternate non-damaging replication programs (Plesa et al., 2006).

In what follows, we shall consider non-cytopathic retroviral interactions; that is, interactions that satisfy α = 1, which is believed to be the case for the equine infectious anemia virus (EIAV) (Schwartz et al., 2018). EIAV shows many characteristics similar to other retroviruses, including a very rapid replication rate and high levels of antigenic variation. It, however, is unusual among retroviruses in that most infected animals, after a few episodes of fever and high viral load, progress to a stage with low viral load and an absence of clinical disease symptoms. The horses effectively control viral replication through adaptive immune mechanisms. Given that this differs from the retroviruses human immunodeficiency virus (HIV) and simian immunodeficiency virus (SIV), in which the infected develop immunodeficiency and disease, EIAV is especially interesting to study in clinical research as well as by using mathematical models. When adopting the mathematical model depicted in Figure 1, the viral clearance rate γ captures these adaptive immune system response mechanisms. In section 2, we shall employ a systematic two-time method (Matkowsky, 1970) to deduce a quasi-steady-state asymptotic closed-form analytic solution of that basic target-cell-limited viral dynamics model.

Although such non-linear problems can be solved numerically the computation must be performed sequentially for each different set of parameter values. The advantage of this asymptotic approach is that it yields an analytic representation, involving the parameters as well as time, required for least-squares parameter-identification curve-fitting procedures to experimental data. We conclude by applying this approach to an experimental data set on EIAV infection.

## 2. The basic target-cell-limited model

The basic model for viral dynamics (see Anderson and May, 1992; Tuckwell and Wan, 2004; Burg et al., 2009; Stancevic et al., 2013) that describes the interactions of a virus with target-cells is given by

(1a) $$\begin{array}{r} \frac{dT}{dt}=\lambda -\rho T-\beta TV \end{array}$$

(1b) $$\begin{array}{r} \frac{dI}{dt}=\beta TV-\delta I \end{array}$$

(1c) $$\begin{array}{r} \frac{dV}{dt}=bI-\gamma V \end{array}$$

where *T* represents the uninfected target-cell population, *I* is the population of infected cells, and *V* is quantity of free virus while *t*, as usual, represents time. It is assumed that the target-cells are produced at a constant rate λ and die at a rate ρ*T*. Free virus infects target-cells at a rate β*TV* and infected cells die at a rate δ*I*. New virus particles are produced at a rate *bI* and are cleared at a rate γ*V*. For the model under consideration, we assume that the viral interaction is non-cytopathic and therefore take ρ = δ in the analysis which follows.

We begin by introducing the dimensionless quantities

$$\begin{array}{l} x\left(\tau ;\varepsilon \right)=\frac{\rho }{\lambda }T\left(t\right),\;y\left(\tau ;\varepsilon \right)=\frac{\rho }{\lambda }I\left(t\right),\;v\left(\tau ;\varepsilon \right)=\frac{\beta }{\rho }V\left(t\right),\;\tau =\rho t,\\ \varepsilon =\rho /\gamma \text{ and }R_{0}=\frac{\lambda \beta b}{\rho \delta \gamma }, \end{array}$$

which upon substitution in Equations (1) yields the dimensionless system

(2a) $$\begin{array}{r} \frac{dx}{d\tau }=1-x-xv \end{array}$$

(2b) $$\begin{array}{r} \frac{dy}{d\tau }=xv-y \end{array}$$

(2c) $$\begin{array}{r} \varepsilon \frac{dv}{d\tau }=R_{0}y-v. \end{array}$$

### 2.1. The method of matched asymptotic expansions

The parameter, ε in Equations (2) is negligible when compared to terms of *O*(1) if the intrinsic death rate of the target-cell population is small when compared to the clearance rate of the virus. We proceed under this assumption and seek a solution of the form

(3) $$\begin{array}{r} \left[x,y,v\right]\left(\tau ;\varepsilon \right)=\left[x_{0},y_{0},v_{0}\right]\left(\tau \right)+O\left(\varepsilon \right). \end{array}$$

Upon substituting Equation (3) into the dimensionless system (Equations 2) and retaining terms of order *O*(1), we obtain the differential-algebraic system

(4a) $$\begin{array}{r} \frac{dx_{0}}{d\tau }=1-x_{0}-x_{0}v_{0} \end{array}$$

(4b) $$\begin{array}{r} \frac{dy_{0}}{d\tau }=x_{0}v_{0}-y_{0} \end{array}$$

(4c) $$\begin{array}{r} v_{0}=R_{0}y_{0}. \end{array}$$

We now construct the inner (or boundary layer) solution, the outer (or quasi-steady-state) solution, and the uniformly valid additive composite.

#### 2.1.1. The inner or boundary layer solution

The presence of ε in Equations (2) suggests that the system contains interactions that occur on two widely different time scales—one fast and one slow. In light of this, we introduce the “transient time” variables

(5) $$\begin{array}{l} \eta =\tau /\varepsilon =\gamma t,\;X\left(\eta ;\varepsilon \right)=x\left(\tau ;\varepsilon \right), \end{array}$$

$$\begin{array}{l} Y\left(\eta ;\varepsilon \right)=y\left(\tau ;\varepsilon \right),\;V\left(\eta ;\varepsilon \right)=v\left(\tau ;\varepsilon \right). \end{array}$$

Upon substituting these into Equations (2) and noting that *d*/*dη* = ε *d*/*dτ* we obtain the boundary layer equations

(6a) $$\begin{array}{r} \frac{dX}{d\eta }=\varepsilon \left(1-X-XV\right), \end{array}$$

(6b) $$\begin{array}{r} \frac{dY}{d\eta }=\varepsilon \left(XV-Y\right), \end{array}$$

(6c) $$\begin{array}{r} \frac{dV}{d\eta }=R_{0}Y-V. \end{array}$$

The ratio of the time scales ε = ρ/γ << 1, is both a consequence of the fact that the virus acts on a fast time scale η = γ*t* and the target-cells, on a slower time scale τ = ρ*t*, and a necessary condition for the employment of a quasi-steady-state approach.

Seeking a solution of Equations (6) of the form

$$\left[x,y,v](\eta ;\varepsilon )=[x_{0},y_{0},v_{0}](\eta )+O(\varepsilon \right)$$

we find that

$$\begin{array}{l} \frac{dX_{0}}{d\eta }=\frac{dY_{0}}{d\eta }=0,\;\frac{dV_{0}}{d\eta }=R_{0}Y_{0}-V_{0}, \end{array}$$

which upon integration yields

(7) $$\begin{array}{l} X_{0}\left(\eta \right)\equiv x^{\left(0\right)},\;Y_{0}\left(\eta \right)\equiv y^{\left(0\right)},\\ V_{0}\left(\eta \right)=R_{0}y^{\left(0\right)}+\left[v^{\left(0\right)}-R_{0}y^{\left(0\right)}\right]e^{-\eta }, \end{array}$$

where *x*^(0)^, *y*^(0)^ and *v*^(0)^ are the *O*(1) values as ε → 0 of the prescribed initial conditions

$$\begin{array}{l} X\left(0;\varepsilon \right)=x^{\left(0\right)},\;Y\left(0;\varepsilon \right)=y^{\left(0\right)},\;V\left(0;\varepsilon \right)=v^{\left(0\right)}. \end{array}$$

#### 2.1.2. The outer solution or the quasi-steady-state approximation

We determine the proper initial conditions to impose for the one-term outer solution functions satisfying Equations (4) by employing the one-term matching rule

$$\begin{array}{l} x_{0}\left(0\right)=\underset{\eta \to \infty }{\lim}X_{0}\left(\eta \right),\;y_{0}\left(0\right)=\underset{\eta \to \infty }{\lim}Y_{0}\left(\eta \right),\;v_{0}\left(0\right)=\underset{\eta \to \infty }{\lim}V_{0}\left(\eta \right), \end{array}$$

which in conjunction with the results of Equation (7) yields

$$\begin{array}{l} x_{0}(0)=x^{\left(0\right)},\;y_{0}(0)=y^{\left(0\right)},\;v_{0}(0)=R_{0}y^{\left(0\right)}, \end{array}$$

where the target-cell initial values can be normalized to satisfy

$$\begin{array}{l} x^{\left(0\right)}+y^{\left(0\right)}=1. \end{array}$$

Since the target-cell populations for both their infected and uninfected states have been non-dimensionalized by employing the same scale factor, this may be accomplished if that common scaling is identified with the initial value of the sum of these populations.

Now returning to Equations (4) and taking the sum of its differential equations, we find that

(8) $$\begin{array}{r} \frac{d\left(x_{0}+y_{0}\right)}{d\tau }+\left(x_{0}+y_{0}\right)=1 \end{array}$$

with initial condition just determined of

(9) $$\begin{array}{r} x^{\left(0\right)}+y^{\left(0\right)}=1. \end{array}$$

Solving this differential equation problem (Equations 8 and 9), we obtain

(10) $$\begin{array}{r} x_{0}\left(\tau \right)+y_{0}\left(\tau \right)\equiv 1\text{ or }y_{0}=1-x_{0}, \end{array}$$

which from Equation(4c) implies

(11) $$\begin{array}{r} v_{0}=R_{0}y_{0}=R_{0}\left(1-x_{0}\right). \end{array}$$

Finally, substituting Equation (11) into Equation (4a) yields the Ricatti equation for *x*_0_ = *x*_0_(τ; *R*_0_):

(12) $$\begin{array}{r} \frac{dx_{0}}{d\tau }=1-\left(R_{0}+1\right)x_{0}+R_{0}x_{0}^{2},\;\tau >0;\;0\leq x_{0}\left(0;R_{0}\right)=x^{\left(0\right)}\leq 1, \end{array}$$

where the initial condition follows from Equation (9). We note that *x*_0_ = 1 is a particular solution of Equation (12), thus we introduce the variable

(13) $$\begin{array}{r} z\equiv x_{0}-1 \end{array}$$

which upon substituting into the above Riccati equation yields the Bernoulli equation

(14) $$\begin{array}{r} \frac{dz}{d\tau }+\left(1-R_{0}\right)z=R_{0}z^{2} \end{array}$$

that can be solved by introducing the variable *w* = *z*^−1^ to obtain

(15) $$\begin{array}{r} z^{-1}=\frac{R_{0}}{1-R_{0}}+ce^{\left(1-R_{0}\right)\tau }. \end{array}$$

Making use of Equation (13) and the initial condition $x_{0}\left(0\right)=x_{i}\equiv x^{\left(0\right)}$, we arrive at the quasi-steady-state approximation for the uninfected target-cell population

(16) $$x_{0}(\tau )=\{\begin{array}{lll} f(\tau )\; & \text{if} & R_{0}=1,\\ g(\tau ) & \text{if} & R_{0}\neq 1, \end{array}$$

where

$$\begin{array}{l} f\left(\tau \right)=\frac{x_{i}+\left(1-x_{i}\right)\tau }{1+\left(1-x_{i}\right)\tau } \end{array}$$

and

$$\begin{array}{l} g\left(\tau \right)=1+\frac{\left(1-R_{0}\right)\left(x_{i}-1\right)}{R_{0}\left(x_{i}-1\right)+\left(1-R_{0}x_{i}\right)e^{\left(1-R_{0}\right)\tau }}. \end{array}$$

**Figure 2:**
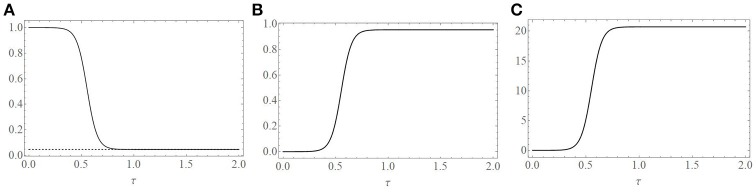
Plots of the uniformly valid additive composite solutions. **(A)** Uninfected cell population, $x_{u}^{\left(0\right)}\left(\tau \right)$, **(B)** infected cell population, $y_{u}^{\left(0\right)}\left(\tau \right)$, and **(C)** free virus population, $v_{u}^{\left(0\right)}\left(\tau \right)$. Populations are expressed as a percent of their initial population values. One dimensionless time unit (τ = 1) corresponds to 21 days. Parameters used to create the plots are given in the text and correspond to *R*_0_ = 21.7 and ε = 0.007.

**Figure 3:**
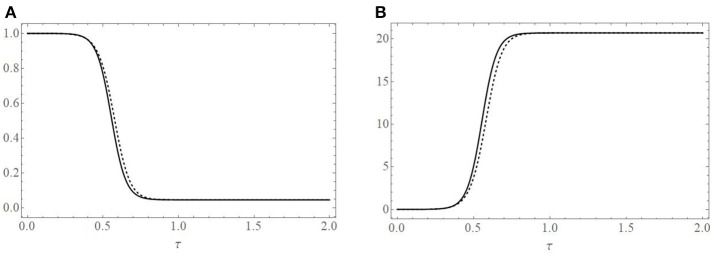
Comparison of the asymptotic solution of the cell population (solid black line), **(A)**, and EIAV population (solid black line), **(B)**, with a numerical simulation (dashed line) of Equations (2). Parameters used to create the plots are given in the text and correspond to *R*_0_ = 21.7 and ε = 0.007.

Note that expressions for *y*_0_(τ) and *v*_0_(τ) follow directly from Equations (10) and (11), respectively. For ease of exposition in what follows we set $y_{i}\equiv y^{\left(0\right)}$ and $v_{i}\equiv v^{\left(0\right)}$. Many similar three-component model systems assume that initially the target-cells are free of the viral infection. If an assumption of that sort were made for our model by taking *y*_*i*_ = 0 or equivalently *x*_*i*_ = 1 then Equation (16) would yield the unrealistic result that *x*_0_(τ) ≡ 1. Hence, we shall approximate that situation by adopting the initial condition *y*_*i*_ = *a* or equivalently *x*_*i*_ = 1−*a* instead where the perturbation infected population density *a* satisfies the condition 0 < *a* << 1. Specifically, for the relevant plots of Figures 2, 3, we shall take *a* = 0.0001 which implies that *x*_*i*_ = 0.9999.

#### 2.1.3. The uniformly valid additive composite

Constructing the one-term uniformly valid additive composites defined by

$$\begin{array}{l} x_{u}^{\left(0\right)}\left(\tau \right)=x_{0}\left(\tau \right)+X_{0}\left(\tau /\varepsilon \right)-x_{i},\\ y_{u}^{\left(0\right)}\left(\tau \right)=y_{0}\left(\tau \right)+Y_{0}\left(\tau /\varepsilon \right)-y_{i},\\ v_{u}^{\left(0\right)}\left(\tau \right)=v_{0}\left(\tau \right)+V_{0}\left(\tau /\varepsilon \right)-R_{0}y_{i}; \end{array}$$

we obtain, from the results of sections 2.1.1 and 2.1.2, that

(18) $$\begin{array}{l} x_{u}^{\left(0\right)}\left(\tau \right)=x_{0}\left(\tau \right),\;y_{u}^{\left(0\right)}\left(\tau \right)=y_{0}\left(\tau \right),\\ v_{u}^{\left(0\right)}\left(\tau \right)=v_{0}\left(\tau \right)+\left[v_{i}-R_{0}y_{i}\right]e^{-\tau /\varepsilon }, \end{array}$$

where

(19) $$\begin{array}{r} y_{0}\left(\tau \right)=1-x_{0}\left(\tau \right)\text{ and }v_{0}\left(\tau \right)=R_{0}y_{0}\left(\tau \right)=R_{0}\left[1-x_{0}\left(\tau \right)\right]. \end{array}$$

Observe, for the target-cell variables, the outer solution is actually uniformly valid to this order.

## 3. Results

In this section we examine the qualitative behavior of the quasi-steady-state approximation given by Equations (18) and (19). We then compare the quasi-steady-state approximation with a numerical simulation of Equations (2) using equine infectious anemia virus (EIAV) data (Schwartz et al., 2018).

From the form of *x*_0_(τ), it is readily seen that when *R*_0_ = 1, *x*_0_(τ) = *f*(τ) → 1 as τ → ∞. If *R*_0_ < 1 then *x*_0_(τ) = *g*(τ) → 1 while if *R*_0_ > 1, *x*_0_(τ) = *g*(τ) → 1/*R*_0_ as τ → ∞, where *x*_0_(τ) is expressed as a percent of its initial population. This is consistent with the global stability results mentioned in section 1.

Figure 2 is a plot of the three uniformly valid composite functions $x_{u}^{\left(0\right)}\left(\tau \right)$, $y_{u}^{\left(0\right)}\left(\tau \right)$, and $v_{u}^{\left(0\right)}\left(\tau \right)$. Parameter values used are median values reported in Schwartz et al. (2018) for the equine infectious anemia virus. Specifically, we take

$$\begin{array}{l} \lambda =2019\text{ cells}/\left(\text{ml }*\text{ day}\right),\\ \beta =3.25\times 10^{-7}\text{ml}/\left(\text{viral RNA copies }*\text{ day}\right),\\ b=505\text{ viral RNA copies}/\left(\text{cell }*\text{ day}\right),\\ \rho =\delta =1/21\text{ per day},\text{ and }\gamma =6.73\text{ per day}. \end{array}$$

Given that a dimensionless time unit (τ = 1) corresponds to 21 days, we see that the uninfected cell population remains relatively constant for approximately 7 days (τ = 0.33). This is followed by a period of eight to ten days of rapid infection of the uninfected cell population at the end of which approximately 95% of the population has been infected by the EIAV.

Figure 3A provides a comparison of the one-term asymptotic representation of the cell population (solid black curve) given by Equation (16) with a numerical simulation (dashed curve) of Equation (2) using the parameter values given above. Figure 3B provides a comparison of the one-term asymptotic representation of the free virus population (solid black curve) with its numerical simulation (dashed curve). The initial virus population was taken to be 450 × β/ρ ≈ 0.00307 viral RNA copies/ml. We note the excellent agreement between the analytic asymptotic representation and numerical simulations.

## 4. Discussion

Researchers that employ the basic viral dynamics model now have an analytic representation involving the parameters that provides a vehicle for least-squares parameter-identification curve-fitting procedures to experimental data. In particular, given a time series population data set ${\left\{\left(t_{n},T_{n}\right)\right\}}_{n=1}^{N}$ and our analytic solution for uninfected target-cells in dimensional variables denoted by *T*(*t*; λ, ρ, β, *b*, γ), a parameter identification residual least squares fit to that data is determined by defining (Torres-Cerna et al., 2016)

$$\begin{array}{l} E\left(\lambda ,\rho ,\beta ,b,\gamma \right)=\overset{N}{\underset{n=1}{\sum }}{\left[T\left(t_{n};\lambda ,\rho ,\beta ,b,\gamma \right)-T_{n}\right]}^{2} \end{array}$$

and minimizing this function by solving for λ_*c*_, ρ_*c*_, β_*c*_, *b*_*c*_, γ_*c*_ such that

$$\begin{array}{l} \frac{\partial E}{\partial \lambda }\left(\lambda_{c},\rho_{c},\beta_{c},b_{c},\gamma_{c}\right)=\frac{\partial E}{\partial \rho }\left(\lambda_{c},\rho_{c},\beta_{c},b_{c},\gamma_{c}\right)\\ =\frac{\partial E}{\partial \beta }\left(\lambda_{c},\rho_{c},\beta_{c},b_{c},\gamma_{c}\right)\\ =\frac{\partial E}{\partial b}\left(\lambda_{c},\rho_{c},\beta_{c},b_{c},\gamma_{c}\right)\\ =\frac{\partial E}{\partial \gamma }\left(\lambda_{c},\rho_{c},\beta_{c},b_{c},\gamma_{c}\right)=0. \end{array}$$

employing the appropriate algorithm. This procedure can be accomplished much more efficiently if one has a closed form representation for *T*(*t*; λ, ρ, β, *b*, γ) as in our case.

We note that for the basic target-cell-limited viral dynamics model, the deduction of an analytic solution for the quasi-steady-state approximation is crucially dependent on the non-cytopathic condition α = δ/ρ = 1 and we have selected parameter values relevant to this scenario for EIAV. If this were the only non-cytopathic virus, our development restricted to the spread of infection in horse populations might not be representative enough to enlist general interest from virologists. Besides EIAV, however, it has been shown that this non-cytopathic assumption is reasonable for a fairly wide class of important viral interactions in human and other animal populations as well, for example, Hepatitis B and C viruses (Wieland and Chisari, 2005). In addition, non-cytopathic enteroviruses such as the coxsackie virus B, one of the agents suspected to be responsible for chronic fatigue syndrome (Landay et al., 1991), cause persistent infections in their host's cells. Another non-cytopathic virus infecting human populations is the Newcastle disease virus (Carver et al., 1967). Finally, Table II in Marcus and Carver (1967) lists a collection of similar non-cytopathic viruses inducing intrinsic interference, among which is the hemadsorption simian virus.

We have been investigating the non-cytopathic interaction of EIAV infection. While similar to human immunodeficiency virus (HIV), EIAV differs from the latter in that it is not fatal, partially because the horses' immune systems help to effectively control the virus. Thus, studies of EIAV infection are of importance since they serve as useful prototypes of viral dynamics and immune control, which may have implications in the development of vaccines for HIV and other retroviral infections.

## Author contributions

RAC led the project, performed model analysis, ran numerical simulations, and wrote the paper. EJS initiated the project, gathered data for application of the model, and assisted with the interpretation of results and writing the paper. DJW introduced the non-cytopathic assumption, performed model analysis, and assisted with writing the paper.

### Conflict of interest statement

The authors declare that the research was conducted in the absence of any commercial or financial relationships that could be construed as a potential conflict of interest.
